# Minimization of dynamic effects in the evolution of dihydrofolate reductase†
†Electronic supplementary information (ESI) available: Full experimental procedures; mass spectra of purified proteins; circular dichroism spectra, tabulated experimental data for *k*_H_, *k*_cat_, and enzyme KIEs, pH dependence of *k*_H_; methodological details of QM/MM calculations including PMFs, recrossing and tunneling coefficients and quantum vibrational corrections. See DOI: 10.1039/c5sc04209g


**DOI:** 10.1039/c5sc04209g

**Published:** 2016-02-03

**Authors:** J. Javier Ruiz-Pernía, Enas Behiry, Louis Y. P. Luk, E. Joel Loveridge, Iñaki Tuñón, Vicent Moliner, Rudolf K. Allemann

**Affiliations:** a Departament de Química Física i Analítica , Universitat Jaume I , 12071 Castelló , Spain . Email: moliner@uji.es; b School of Chemistry & Cardiff Catalysis Institute , Cardiff University , Park Place , Cardiff , CF10 3AT , UK . Email: allemannrk@cf.ac.uk; c Departament de Química Física , Universitat de València , 46100 Burjassot , Spain . Email: ignacio.tunon@uv.es

## Abstract

Protein isotope labeling is a powerful technique to probe functionally important motions in enzyme catalysis and can be applied to investigate the conformational dynamics of proteins. Previous investigations have indicated that dynamic coupling is detrimental to catalysis by dihydrofolate reductase (DHFR) from the mesophile *Escherichia coli* (EcDHFR). Comparison of DHFRs from organisms adapted to survive at a wide range of temperatures suggests that dynamic coupling in DHFR catalysis has been minimized during evolution; it arises from reorganizational motions needed to facilitate charge transfer events. Contrary to the behaviour observed for the DHFR from the moderate thermophile *Geobacillus stearothermophilus* (BsDHFR), the chemical transformation catalyzed by the cold-adapted bacterium *Moritella profunda* (MpDHFR) is only weakly affected by protein isotope substitutions at low temperatures, but the isotopically substituted enzyme is a substantially inferior catalyst at higher, non-physiological temperatures. QM/MM studies revealed that this behaviour is caused by the enzyme’s structural sensitivity to temperature changes, which enhances unfavorable dynamic coupling at higher temperatures by promoting additional recrossing trajectories on the transition state dividing surface. We postulate that these motions are minimized by fine-tuning DHFR flexibility through optimization of the free energy surface of the reaction, such that a nearly static reaction-ready configuration with optimal electrostatic properties is maintained under physiological conditions.

## Abstract 2

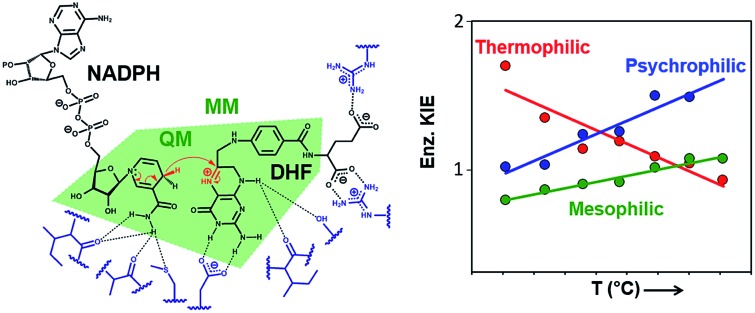
Protein isotope labeling is a powerful technique to probe functionally important motions in enzyme catalysis and can be applied to investigate the conformational dynamics of proteins.

## Introduction

Exploring the energy landscape of enzyme catalysis is a theme central to several areas of research. While it has long been known that protein conformational changes and flexibility are critical for the progression of the physical steps of enzyme catalytic cycle,^1^–^5^ the role of protein motions in formation of the transition state of enzyme-catalyzed reactions, and the molecular mechanism involved, have not been fully elucidated.^6^–^18^ Some authors suggest dynamics as a key driving force in catalysis of the chemical step of an enzyme catalyzed reaction, but others have shown that reduction of the activation free energy – an equilibrium property – is the source of catalysis.^6^–^14^ In recent years, ‘enzyme isotope effects’ have been developed as a powerful tool to probe the nature of enzyme motions and their response to changes in the reaction conditions.^8^,^19^–^30^ In such studies, the kinetics of ‘heavy’ enzymes, isotopically labeled at non-exchangeable positions (*e.g.*^2^H, ^13^C and ^15^N), are compared with those of the ‘light’ counterparts with natural abundance isotopes. The coupling of protein motions to enzyme catalysis is revealed as a difference between the kinetic properties of the isotopologous enzymes, because mass-dependent translational, vibrational and rotational motions are altered by heavy isotope substitution, whereas the potential energy surface and electrostatic properties are unaffected.^19^,^20^ More recently, this method has been extended to probe the dynamic contributions of individual regions of an enzyme,^29^ or even individual residue types.^30^

On the basis of protein isotope labeling studies, dynamic coupling on the femtosecond timescale has been proposed for a number of enzymes under or near physiological conditions.^8^,^19^–^26^,^28^,^30^–^32^ In this work, we reserve ‘dynamics’ to mean fast, non-stochastic protein vibrations, while ‘motions’ refers to equilibrium fluctuations on longer timescales. It should be noted that kinetic analyses by protein isotope labeling can report on both, depending on the type of kinetic measurement employed. For two of the best-studied systems, purine nucleoside phosphorylase (PNP) and dihydrofolate reductase (DHFR), dynamic coupling has been postulated to be beneficial in the former^30^–^32^ but is detrimental in the latter.^8^,^25^–^29^ To explain these apparently contradictory observations, it is useful to compare the effects of protein dynamics on the reaction coordinate between enzyme homologues that have evolved in different ecological niches to catalyze the same reaction under different physiologically optimal conditions.

**Fig. 1:**
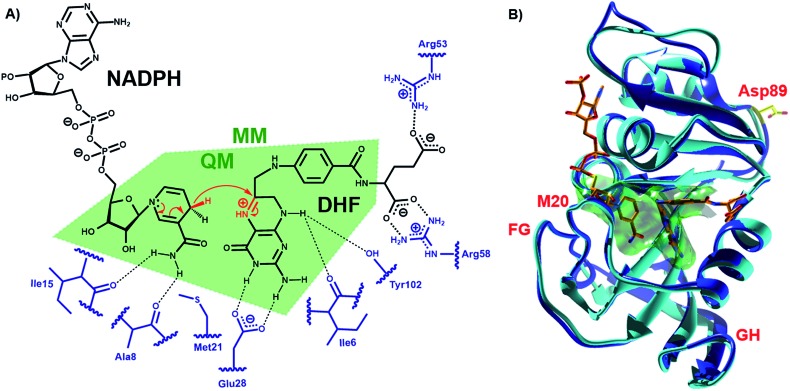
(A) Schematic representation of the active site and the DHFR-catalyzed reaction. (B) Alignment of the cartoon structures of MpDHFR (blue, PDB 2ZZA) and EcDHFR (cyan, PDB ; 1RX2)^34^ in complex with NADP^+^ and folate. In both panels, the QM/MM subsystem is shaded green.

DHFR, which catalyzes the transfer of the pro-*R* hydride of NADPH to C-6 and a solvent proton to N-5 of dihydrofolate (DHF) (Fig. 1), has become a paradigmatic model to investigate the influence of protein motions on enzyme catalysis.^7^–^12^,^24^,^25^,^33^–^46^ Previously, the reaction kinetics for DHFRs from the mesophile *Escherichia coli* (EcDHFR), the thermophile *Geobacillus stearothermophilus* (BsDHFR) and the hyperthermophile *Thermotoga maritima* (TmDHFR) have been analyzed by protein isotope labeling.^8^,^24^–^29^ In all cases, dynamic coupling was found to be insignificant under physiological conditions. However, the enzyme isotope effect increases mildly with temperature in EcDHFR,^25^,^29^ while for BsDHFR the effect is stronger at low temperatures,^28^ and the hydride transfer rate constants for TmDHFR are unaffected by isotopic substitution of the enzyme at all temperatures examined.^27^ QM/MM analyses confirmed that protein dynamics couple to the reaction coordinate, defined as a function of the bonds that are being formed and broken, and increase the unfavorable recrossing trajectories on the transition state dividing surface defined for that coordinate.^8^,^25^,^26^,^28^,^29^ Depending on the frequencies associated with protein motions, these may or may not be in equilibrium with the reaction coordinate, being incorporated in the evaluation of the rate constant either in the activation free energy or in the recrossing transmission coefficient, respectively. The presence of non-equilibrium dynamics reduces the recrossing transmission coefficient to values below unity, diminishing the rate constant. This effect can be described as an effective friction acting on the motion along the reaction coordinate.^47^ In this picture, recrossings of the dividing surface are linked to the participation of protein dynamics in the barrier crossing event. The magnitude of the enzyme isotope effect increases when the enzyme lacks either the thermal energy^28^ or the conformational flexibility^26^ needed to adopt a configuration from which the barrier to hydride transfer may be surmounted. In effect, dynamic coupling can be viewed as a non-equilibrium enzyme reorganization in response to the electronic rearrangement that occurs at the top of the free energy barrier, and the degree of reorganization required is affected by the same factors that dictate the level of equilibrium preorganization (and hence equilibrium reorganization required to stabilize the transition state as the barrier is climbed). Moreover, heavy isotopic labeling of isolated segments of EcDHFR revealed that dynamic coupling does not necessarily originate from the mobile loops of the enzyme, even though the equilibrium motions of these regions are critical for the physical steps of catalytic turnover.^29^ Instead, dynamic coupling in DHFR appears to arise when reorganizational motions are required to overcome an incomplete electrostatic preorganization of the active site.^8^,^29^ As the (equilibrium) preorganization process is naturally optimized (although not perfected) during the evolution of most enzymes, dynamic coupling should be minimized under physiological conditions but may be enhanced when conditions become sub-optimal. The temperature dependence of the enzyme kinetic isotope effects for monomeric DHFRs from psychro-, meso- and thermophilic organisms should therefore be predictable. To test this hypothesis the DHFR from *Moritella profunda* (MpDHFR), a cold-adapted organism that thrives at temperatures below 5 °C, was investigated (Fig. 1).

MpDHFR has a melting temperature of only 38 °C (ref. 48) (compared to 52 °C for EcDHFR^49^) and is generally more flexible than EcDHFR.^41^ Cold adaptation mostly arises from the introduction of solvent-exposed hydrophobic residues and partly by the removal of proline residues.^50^ The core regions of EcDHFR and MpDHFR are highly similar and most of the amino acid substitutions are found on the enzyme surface.^50^ Furthermore, despite its increased flexibility and contrary to EcDHFR, which cycles through the closed and occluded conformations,^4^,^34^ MpDHFR does not appear to undergo major conformational change during progression through the reaction cycle.^51^,^52^ Here, we report the kinetic properties of ‘light’ and ‘heavy’ MpDHFR. The MpDHFR kinetic isotope effect is minimal at physiological temperature, but in contrast to other non-psychrophilic DHFR homologues, its magnitude increases sharply with temperature. Based on our experimental and computational analyses, this trend appears to be intrinsically linked to the structural properties of the reaction-ready configuration of the enzyme, the integrity of which is highly temperature-dependent in MpDHFR.

## Results and discussion

### Creation of ‘heavy’ MpDHFR

Minimal media containing the appropriate isotopically labeled nutrients were used to generate perdeuterated, ^13^C, ^15^N doubly labeled, and ^13^C, ^15^N, ^2^H triply labeled MpDHFRs (see ESI†). According to mass spectrometric analyses (Fig. S1†) there was a 10.5% molecular weight (MW) increase for the triply labeled (‘heavy’) enzyme, while both the perdeuterated and ^13^C, ^15^N labeled enzymes showed approximately a 5.7% MW increase (in this article the term ‘heavy’ enzyme refers to the triply labeled enzyme only). Over 99% of the non-exchangeable atoms in these enzymes were substituted with the corresponding isotopic labels. Enzyme purification and all kinetic measurements were performed in buffered H_2_O so that protons replaced exchangeable ^2^H.

### Experimental results: steady-state turnover

MpDHFR has the same kinetic cycle as EcDHFR, and at pH 7.0 the turnover rate is limited by product release.^52^ Steady-state turnover rate constants *k*LEcat for the ‘light’ enzyme (MpDHFR with natural abundance isotopic distribution) are noticeably higher than those for the ‘heavy’ enzyme (*k*HEcat) (Table S1†), giving an enzyme kinetic isotope effect KIE_cat_ (*k*LEcat/*k*HEcat) that decreased gradually from 2.02 ± 0.29 at 5 °C to 1.47 ± 0.11 at 30 °C (Fig. S2 and Table S2†). As protein isotope labeling leads to KIEs that report on the involvement of protein motions rather than the catalyzed chemistry *per se*, a steady-state enzyme KIE_cat_ is not unexpected. Perdeuterated and ^15^N, ^13^C doubly labeled MpDHFRs showed identical values for the enzyme KIE_cat_ and its temperature dependence. The Michaelis constants for NADPH and DHF are essentially the same for all these enzymes, suggesting that binding of starting materials was not affected by enzyme isotope labeling (Table S3†).

### Experimental results: the chemical step

**Fig. 2:**
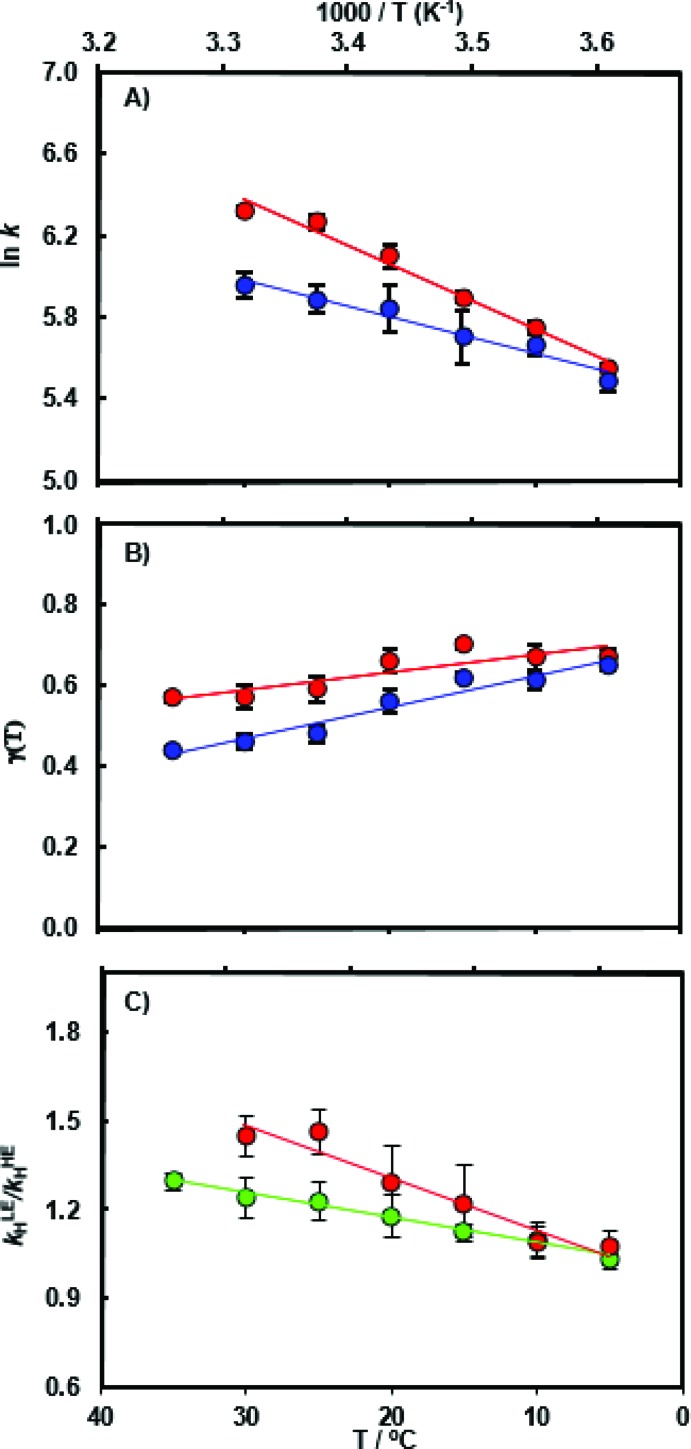
(A) Arrhenius plot of the experimental pre-steady state rate constants *k*_H_ during catalysis by light MpDHFR (red) and its heavy counterpart (blue), and temperature dependence of (B) the corresponding recrossing coefficients, and (C) the resulting enzyme KIE (*k*LEH/*k*HEH) calculated experimentally (red) and computationally (green).

The chemical transformation for the MpDHFR catalyzed reaction was characterized at pH 7.0 in pre-steady-state stopped flow experiments. At the physiologically relevant temperature of 5 °C, the hydride transfer rate constant of the ‘light’ enzyme (*k*LEH) is close to that of the ‘heavy’ enzyme (*k*HEH) (Table S1†) resulting in an enzyme kinetic isotope effect KIE_H_ of 1.09 ± 0.04. With rising temperature the value for the enzyme KIE_H_ increases noticeably and reaches 1.47 ± 0.04 at 30 °C (Fig. 2 and Table S3†). While the experimental data at 30 °C may suggest the beginning of thermal denaturation (as supported by our computational results discussed below), omitting these data did not significantly alter the activation parameters obtained from fitting the data to the Eyring equation, but did reduce the quality of the fits. Perdeuterated and ^15^N, ^13^C doubly labeled MpDHFRs reproduced essentially the same magnitude and temperature dependence of the enzyme KIE_H_, demonstrating that the isotope effects are unlikely to be caused by alteration of the van der Waals volume of the enzyme due to deuterium labeling. Also, the p*K*_a_ of the hydride transfer reaction remains unchanged upon heavy isotope substitution and was ∼6.2 in both the ‘light’ and the ‘heavy’ enzyme (Fig. S5 and Table S4†). At higher, non-physiological temperatures, enhanced dynamic coupling to the reaction coordinate is therefore the likely cause of the decreased hydride transfer rate constants in the ‘heavy’ enzyme. This supports our proposal that at non-physiological temperatures where electrostatic preorganization of the substrates is not optimal and additional equilibrium reorganizational motions are required during the formation of the transition state, non-equilibrium dynamic coupling also becomes more pronounced.^26^,^28^,^39^,^53^

### Computational results

**Fig. 3:**
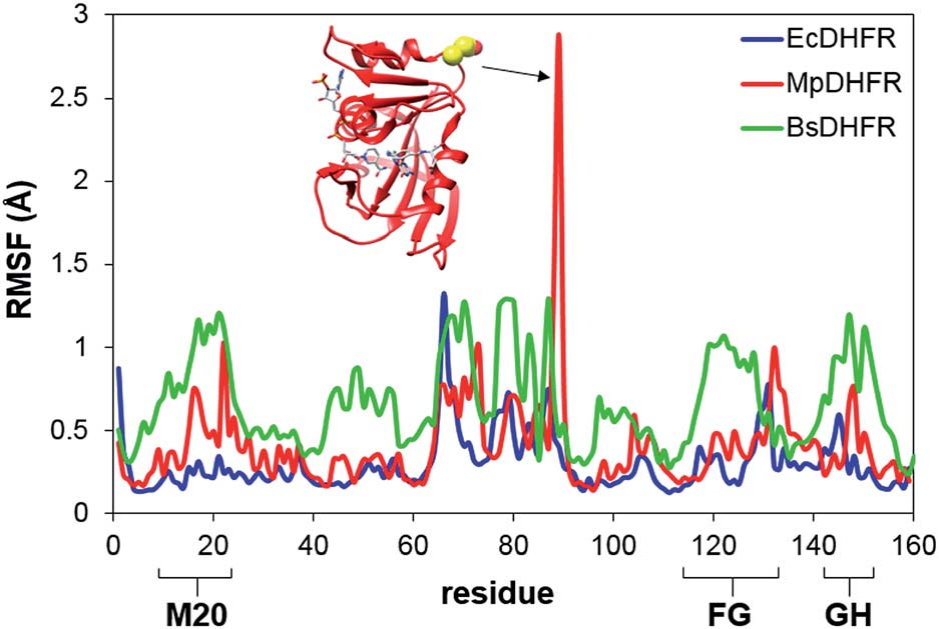
RMSFs obtained at 298 K for MpDHFR, BsDHFR and EcDHFR in the reactant state. The inset indicates the positioning of residue Asp89 which displays a large value of the RMSF in MpDHFR.

To analyze the catalytic pathway of the MpDHFR reaction, QM/MM molecular simulations of this enzyme were carried out based on previously established procedures.^25^,^26^,^28^,^29^ As shown in Fig. 1, the quantum subsystem contained the nicotinamide ring and the ribose of the cofactor, and the pteridine ring and the *N*-methylene-substituted *p*-aminobenzoyl moiety, pABA, of the substrate. The rest of the system was treated by MM force fields (for details see ESI†). The Root-Mean-Squared Fluctuation (RMSF) for each residue was evaluated by running 5 ns MD simulations of the equilibrated reactant state at 298 K (Fig. 3). The flexibility of MpDHFR was found to be between that of EcDHFR and BsDHFR, as suggested by the enzyme KIE_cat_ (*vide supra*). Compared to EcDHFR, the RMSF values corresponding to the M20, FG and GH loops (residues 9–23, 116–132 and 142–149, respectively) (Fig. 1) are slightly greater in MpDHFR. Moreover, Asp89 of MpDHFR, located within the hinge region connecting the adenosine binding and loop domains, is noticeably more flexible than the corresponding residue in EcDHFR and BsDHFR (Fig. 1 and 3). Dynamic cross correlation maps^54^ do not show qualitative differences between the three enzymes, although more correlations between residues are observed for BsDHFR (Fig. S8†).

The rate constants of ‘light’ and ‘heavy’ MpDHFR were evaluated under the framework of Ensemble Averaged Variational Transition State Theory (EA-VTST), which was corrected for tunneling contributions and dynamic effects:^55^–^57^
1
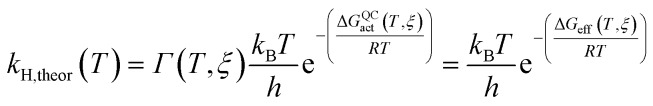
where *R* is the ideal gas constant, *T* is the temperature, *k*_B_ is the Boltzmann constant, *h* is Planck's constant, Δ*G*_eff_ is the effective activation free energy, which includes all the contributions to the rate constant and can be readily compared to the value derived from the experimental rate constant. Δ*G*QCact is the quasiclassical activation free energy calculated along the reaction coordinate *ξ*:^58^

2 Δ*G*QCact(*T*, *ξ*) = Δ*G*CMact(*T*, *ξ*) + Δ*G*QMvib(*T*)

where Δ*G*CMact(*T*, *ξ*) is the activation free energy obtained from the classical Potential of Mean Force (PMF) along the selected reaction coordinate and Δ*G*QMvib(*T*) is a correction term due to the quantized nature of molecular vibrations (mainly zero-point energies).^58^–^61^ In eqn (1), *Γ*(*T*, *ξ*) is the temperature-dependent transmission coefficient that contains dynamic and tunneling corrections to the classical rate constant:

3 *Γ*(*T*, *ξ*) = *γ*(*T*, *ξ*)*κ*(*T*)

where *γ*(*T*, *ξ*) is the recrossing transmission coefficient that corrects the rate constant for the trajectories that recross the dividing surface from the product valley back to the reactant valley, and *κ*(*T*) is the tunneling coefficient that accounts for reactive trajectories that do not reach the classical threshold energy. The enzyme KIEs were calculated from the ratio of the corresponding transmission coefficients computed for the light and heavy enzymes (*vide infra*). In our QM/MM simulations, the reaction coordinate is the antisymmetric combination of the distances of the hydride to the donor and to the acceptor atoms and does not depend on the coordinates of the protein (for details see ESI†). Any non-equilibrium influence of protein dynamics (revealed as a variation of the rate constant due to the vibrational shift of protein motions caused by mass substitution) should therefore be captured in the transmission coefficient (Table S5†).^28^ Theoretically, it is still possible that variables other than the transmission coefficients can be affected by heavy isotope substitution, although our previous estimations of other DHFRs have provided accurate predictions of enzyme KIEs.^25^,^26^,^28^,^29^ Hence, the effect of protein isotope labeling on most of the parameters was analyzed within this procedure.

The classical PMF was computed to obtain the quasi-classical activation free energies after quantum corrections of vibrational coupling (eqn (2), Table 1). The reactant state averaged geometries at various temperatures (278, 298 and 308 K) were obtained from the windows corresponding to the maximum and minimum of the PMF (Fig. 1, S6 and Table S6†). The onset of thermal denaturation is evident from the changes of the reactant state structure (Table S7†), in which the interactions between the amide group of the cofactor and residues of the M21 loop in MpDHFR (M20 in EcDHFR) are weakened at 308 K (dHN1_cof_-O_Ile15_, dHN2_cof_-O_Ile15_ and dHN2_cof_-S_Met21_). Previous studies have indicated that protein isotope labeling has a negligible effect on the electrostatic potentials of the enzyme.^28^ Consequently, the force fields of ‘light’ and ‘heavy’ MpDHFR and their classical activation free energy barriers Δ*G*CMact(*T*, *ξ*) were identical. Residues up to 6 Å from the substrate or cofactor were incorporated in the calculation of the Hessian to include the mass modification effect into the tunneling prefactor (*κ*) and vibrational corrections (Δ*G*QMvib(*T*)). Similar to previous studies,^25^,^26^,^28^ the tunneling coefficients of the ‘light’ and ‘heavy’ MpDHFR are statistically identical (Table 1). Protein isotope substitution affects the zero point energies of the transition state and reactants to a different extent, thus there is a slight change in the corresponding Δ*G*QMvib(*T*) values (Table 1). However, this difference is too small to account for the experimental enzyme KIE_H_, which is particularly strong at higher temperatures. Instead, the only significant difference found in the calculations of the ‘light’ and ‘heavy’ MpDHFR is in their recrossing coefficients. Accordingly, the enzyme KIE_H_ can be approximated as follows:
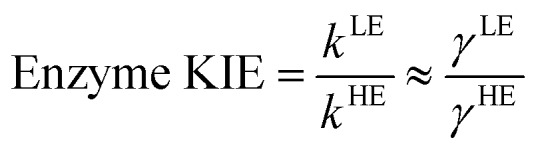


**Table 1:** Contributions to the TST rate constant at 298 K due to recrossing (*γ*) and tunneling (*κ*), classical free energy barrier (Δ*G*CMact(*T*, *ξ*)), vibrational corrections (Δ*G*QMvib(*T*)) quasi-classical free energy of activation (Δ*G*QCact) and effective phenomenological free energies of activation (Δ*G*_eff_) determined by QM/MM calculations

MpDHFR	*γ*	*κ*	Δ*G*QMvib(*T*) (kcal mol^–1^)	Δ*G*CMact(*T*, *ξ*) (kcal mol^–1^)	Δ*G*QCact (kcal mol^–1^)	Δ*G*_eff_ (s^–1^)	Exp. Δ*G*^‡^ (kcal mol^–1^)
Light	0.59 ± 0.03	3.5 ± 0.5	–1.35 ± 0.08	12.7 ± 1.0	11.3 ± 1.0	10.9 ± 1.0	13.8 ± 0.1
Heavy	0.48 ± 0.02	3.4 ± 0.6	–1.28 ± 0.08		11.4 ± 1.0	11.1 ± 1.0	13.9 ± 0.2

To evaluate the temperature dependence of the enzyme KIE_H_, transmission coefficients of ‘light’ and ‘heavy’ MpDHFR were extracted by locating the positions of the TS in 7 separate QM/MM simulations of different temperatures (*T* = 278, 283, 288, 293, 298, 303 and 308 K). It should be noted that the magnitudes of the transmission coefficients depend on the selection of the reaction coordinate, which is defined by the coordinates of the substrate and cofactor but not the enzyme itself. Consequently, this strategy provides precise characterization of the mass-induced environmental effects and the transmission coefficients can be retrieved with minimal statistical errors.

As anticipated, the recrossing coefficient *γ* is most sensitive to protein isotope labeling (Fig. 2B and Table S5†). There is a sharp increase in the computational enzyme KIE_H_ with increasing temperature, which resembles the experimental observations, and a noticeably larger magnitude of δ*γ*/δ*T* than those observed for Ec- and BsDHFR (Table 2). For the moderately thermophilic BsDHFR, which possesses relatively high sequence homology to MpDHFR (38%; Table S8†), the enzyme KIE_H_ decreased with increasing temperature, while EcDHFR showed a slight increase in enzyme KIE_H_ with increasing temperature. This suggests that there are various biophysical factors that change the frequency of recrossing events in DHFR. If non-equilibrium dynamic coupling is controlled by the same factors as equilibrium protein reorganizational motions involved in charge transfer events along the reaction coordinate,^8^,^29^ then both equilibrium and non-equilibrium motions should normally be minimal. The enzyme provides an electrostatic and geometric environment complementary to the transition state,^8^ and electrostatic preorganization in the chemical step is optimized relative to water. However, since enzymes are relatively flexible biomolecules and increasing temperature activates thermal motions, additional friction is expected to be incorporated into the reaction coordinate at higher temperatures, resulting in a negative δ*γ*/δ*T*. Likewise, protein denaturation at higher temperature will also lead to a negative δ*γ*/δ*T*, as structural integrity is lost with increasing temperature. On the other hand, the efficiency of conformational sampling and other critical equilibrium thermal processes also depend on the intrinsic flexibility of the enzyme. Their relationship is demonstrated in BsDHFR, where protein rigidification at low temperature has a stronger effect than denaturation or increased friction, causing an increase of recrossing events, and hence the enzyme KIE_H_, with increasing temperature.^28^

**Table 2:** The change of recrossing coefficients with respect to temperature (δ*γ*/δ*T*) and experimental Eyring activation parameters of the light and heavy DHFRs at pH 7.0 under pre-steady state conditions at 25 °C

	MpDHFR		TmDHFR^a^		BsDHFR^b^		EcDHFR^c^	
	Light	Heavy	Light	Heavy	Light	Heavy	Light	Heavy
d*γ*/d*T*	–0.0044	–0.0076	N/A	N/A	–0.0024	0.0026	–0.0014	–0.0033
Δ*S*^‡^ (kcal mol^–1^ K^–1^)	–30 ± 1	–39 ± 1	–23 ± 1	–23 ± 1	–27 ± 2	–21 ± 2	–26 ± 1	–30 ± 2
Δ*H*^‡^ (kcal mol^–1^)	4.7 ± 0.2	2.4 ± 0.2	11.7 ± 0.1	11.7 ± 0.1	6.5 ± 0.3	8.4 ± 0.6	6.7 ± 0.3	5.4 ± 0.6
Δ*G*^‡^ (kcal mol^–1^)	13.8 ± 0.1	13.9 ± 0.2	18.4 ± 1.3	18.4 ± 1.9	14.6 ± 1.6	14.7 ± 1.8	14.4 ± 1.5	14.4 ± 2.5

In MpDHFR, an enzyme KIE_H_ of close to 1 at low temperature implies that this cold-adapted enzyme has sufficient flexibility to sample an ideal configuration conducive for hydride transfer, even when the thermal energy of the surroundings is relatively low. However, cold adaptation of MpDHFR means that thermal denaturation is likely to be the dominant factor in determining the degree of dynamic coupling. According to the RMSF analysis described above and previous results,^48^,^49^,^62^ the hinge near the adenosine-binding region (residues 87–89) is highly flexible on both the ms and ns timescales. In EcDHFR, this position has been shown to lose its native structure early in the thermal unfolding process.^49^,^62^ This therefore renders MpDHFR particularly sensitive to thermal degradation, and the integrity of the reaction-ready configuration collapses rapidly with increasing temperature. In addition, circular dichroism spectra of MpDHFR show a gradual decrease in structural integrity with increasing temperature rather than a sharp loss of secondary structure.^48^ Recrossing dynamics therefore increase progressively in this enzyme, giving the most negative value of δ*γ*/δ*T* among DHFRs. Since enhanced recrossing dynamics increase the activation entropy,^28^ the magnitude of Δ*S*^‡^ increases most strongly for MpDHFR upon heavy isotope substitution compared to all other DHFRs examined so far (Table 2).

Following this argument, protein flexibility and thermal integrity of the reaction-ready configuration in EcDHFR appear to be well balanced, such that the reaction can proceed efficiently in a mesophilic environment with minimal impact from dynamic coupling. Our results are in broad agreement with other frameworks, in which wild type enzymes have well-organized active sites under physiological conditions, with deviations from these optimized conditions increasing the need for distance sampling (reorganization) along the reaction coordinate.^6^,^63^,^64^ The EcDHFR-N23PP/S148A variant is a good example: intrinsic isotope effect measurements suggest that distance sampling is necessary in the variant but not in wild type EcDHFR,^65^ and protein isotope labeling similarly suggests that dynamic coupling is minimal in the wild type enzyme but becomes more pronounced in the variant.^26^ However, distance sampling is an equilibrium process under direct control of the free energy surface, whereas dynamic coupling need not be. Our protein isotope labeling results are also consistent with an epistatic network of residues that are functionally important but not directly involved in catalyzing the chemical step,^6^,^36^,^66^ as this network may be involved in stability, folding, or even the initial preorganization of the active site for catalysis. Although δ*γ*/δ*T* in TmDHFR has not been determined, it is likely independent of protein isotope labeling since the corresponding enzyme KIE is close to unity for all temperatures. Given that TmDHFR is highly rigid,^67^–^69^ it is reasonable to expect that protein reorganizational motions are minimal even though the electrostatic environment of the active site is not optimal, but additional computational analyses are needed to confirm this. In all of these DHFRs, the experimental activation free energies for the ‘light’ and ‘heavy’ enzymes are statistically the same due to enthalpy/entropy compensation (Table 2). One plausible explanation for this observation is that the magnitudes of Δ*H*^‡^ and Δ*S*^‡^ correlate to the efficiency of conformational sampling and the stability of the reaction-ready configuration; the former depends greatly on the intrinsic flexibility of the enzyme whereas the latter requires a certain degree of protein rigidity.

## Conclusions

Studies with a number of DHFRs suggest that dynamic coupling is minimized under physiological conditions^8^,^24^–^29^ and hence that it has been selected against during evolution of DHFRs. This interpretation ties in with other studies that suggest wild type enzymes to have well-organized active sites under physiological conditions,^6^,^23^,^44^ and supports a view of DHFR catalysis in which electrostatic effects play a dominant role.^7^,^9^,^39^,^53^,^70^,^71^ Since DHFR catalysis is generally limited by a physical step in the reaction cycle rather than the chemistry itself, dynamic coupling is mostly likely linked to equilibrium processes that are under strong selective pressure, in the sense that factors that control these equilibrium processes also affect the degree of non-equilibrium dynamic coupling. Given that DHFR catalyzes a relatively simple reaction that involves only few charge transfer events, we hypothesize that dynamic coupling arises from reorganizational motions that are spontaneously minimized upon efficient conformational sampling, an equilibrium process that is directly linked to the nature of enzyme flexibility and therefore the free energy surface. For an enzyme to function optimally, its flexibility needs to be finely tuned so that both equilibrium and non-equilibrium processes can proceed in a favorable manner. Results for DHFR are at odds with those reported previously for PNP that have been interpreted to suggest that dynamic coupling is beneficial.^30^–^32^ To further explore the role of dynamic coupling, its effects on the chemical reactions catalyzed by other enzymes must therefore be investigated. Such work may eventually facilitate the design of artificial enzymes with nature-like rate accelerations.

## Supplementary Material

Supplementary information
