# Supplementary material for: Minimization of dynamic effects in the evolution of dihydrofolate reductase
Source: Chem Sci. 2016 Feb 3;7(5):3248–55. doi: 10.1039/c5sc04209g (PMC6006479; doi:10.1039/c5sc04209g)
Supplement: Supplementary file 1 [file SC-007-C5SC04209G-s001.pdf]

Electronic Supplementary Material (ESI) for Chemical Science.
This journal is © The Royal Society of Chemistry 2016

Electronic Supplementary Information for

Minimization of dynamic effects in the evolution of dihydrofolate reductase

J. Javier Ruiz-Pernia[a,†], Enas Behiry[b,†], Louis Y. P. Luk[b], E. Joel Loveridge[b], Iñaki Tuñón[c,2,*], Vicent Moliner[a,2,*], Rudolf K. Allemann[b,2,*]

[a.] Departament de Química Física i Analítica, Universitat Jaume I, 12071 Castelló, Spain
[b.] School of Chemistry & Cardiff Catalysis Institute, Cardiff University, Park Place, Cardiff, CF10 3AT, UK
[c.] Departament de Química Física, Universitat de València, 46100 Burjassot, Spain
[†] These authors contribute equally.

Experimental work

Materials. ^{15}N-ammonium chloride, [$^{13}C_6$, $^{2}H_7$]-glucose, 99.9% $^{2}H_2O$ and folate were purchased from Sigma-Aldrich. NADPH was purchased from Melford. Dihydrofolate was prepared by dithionite reduction of folate.[1] The concentration of NADPH was determined spectrophotometrically using an extinction coefficient of 6,200 M^{-1} cm^{-1} at 339 nm, and an extinction coefficient of 28,000 M^{-1} cm^{-1} was used to determine the concentration of DHF at 282 nm.

Enzyme preparation. The gene encoding MpDHFR, optimized for expression in *Escherichia coli* and cloned into a pET-21a(+) expression vector, was purchased from Genescript®. BL21 Star (DE3) competent cells were transformed with this plasmid. Gene expression in minimal media containing the appropriate isotopically labeled ingredients, and protein purification by both Q-sepharose and Superdex chromatography, were performed as described previously.[2,3] Electrospray ionization mass spectrometry (ESI-MS) indicated masses of 18292 ± 4, 19310 ± 1, 19235 ± 1 and 20214 ± 7 for light, perdeuterated, ^{13}C- and ^{15}N-doubly labeled and ^{2}H-, ^{13}C-, ^{15}N-triply labeled heavy enzymes, respectively (Figure S1).

Steady state kinetic measurements. Catalytic turnover rate constants were measured spectrophotometrically on a Shimadzu spectrophotometer by following the decrease in absorbance at 340 nm during the reaction (ε_{340} = 11,800 M^{-1} cm^{-1}).[2,3] Initial rates were determined under saturating conditions (100 μM NADPH and DHF) at pH 7.0 in 100 mM potassium phosphate containing 100 mM NaCl and 10 mM β-mercaptoethanol, and Michaelis constants were measured at pH 7.0 at 20 °C and 5 °C in 100 mM potassium phosphate containing 100 mM NaCl and 10 mM β-mercaptoethanol. To avoid hysteresis,[3] the enzyme (20-50 nM) was pre-incubated at the desired temperature with NADPH (0.1-200 μM) for 1 min prior to addition of DHF (0.1-100 μM). When the concentration of NADPH was varied, that of DHF was maintained at 100 μM and vice versa. Each data point is the result of three independent measurements. The change in initial rate with substrate concentration was fit to the Michaelis-Menten equation using SigmaPlot 10.

Pre-steady state kinetic measurements. Hydride transfer rate constants were measured under single-turnover conditions on an Applied Photophysics stopped-flow spectrophotometer. The enzyme (20 μM final concentration) was pre-incubated with NADPH (10 μM final concentration) for at least 5 min in 100 mM potassium phosphate (pH 7.0) containing 100 mM NaCl and 10 mM β-mercaptoethanol, and the reaction was started by rapidly mixing with DHF (200 μM final concentration) in the same buffer. Increasing the concentration of DHF to 400 μM does not affect the rate constants. Loss of fluorescence resonance energy transfer from the enzyme to NADPH during the reaction was observed by exciting the sample at 292 nm and measuring the emission using an output filter with a 400 nm cut-off, and double exponential fitting was applied. Each data point was an average of four to six measurements. This was repeated three times and errors were propagated. To determine the pK_a of the hydride transfer reaction catalyzed by both light and heavy MpDHFRs, pre-steady state kinetics were measured in MTEN buffer (50 mM MES, 25 mM Tris, 25 mM ethanolamine, 100 mM NaCl and 10 mM β-mercaptoethanol) at 5° C. Measurements at higher temperatures were limited by the extremely fast reaction at low pH. Activation parameters were obtained by fitting the data for each enzyme to the Eyring equation: $\ln\frac{k}{T} = -\frac{\Delta H^{\ddagger}}{RT} + \frac{\Delta S^{\ddagger}}{R} + \ln\frac{k_B}{h}$, where k is rate constant, $\Delta H^{\ddagger}$ is activation enthalpy,. $\Delta S^{\ddagger}$ is activation entropy, k_B is Boltzmann constant and h is Planck's constant.

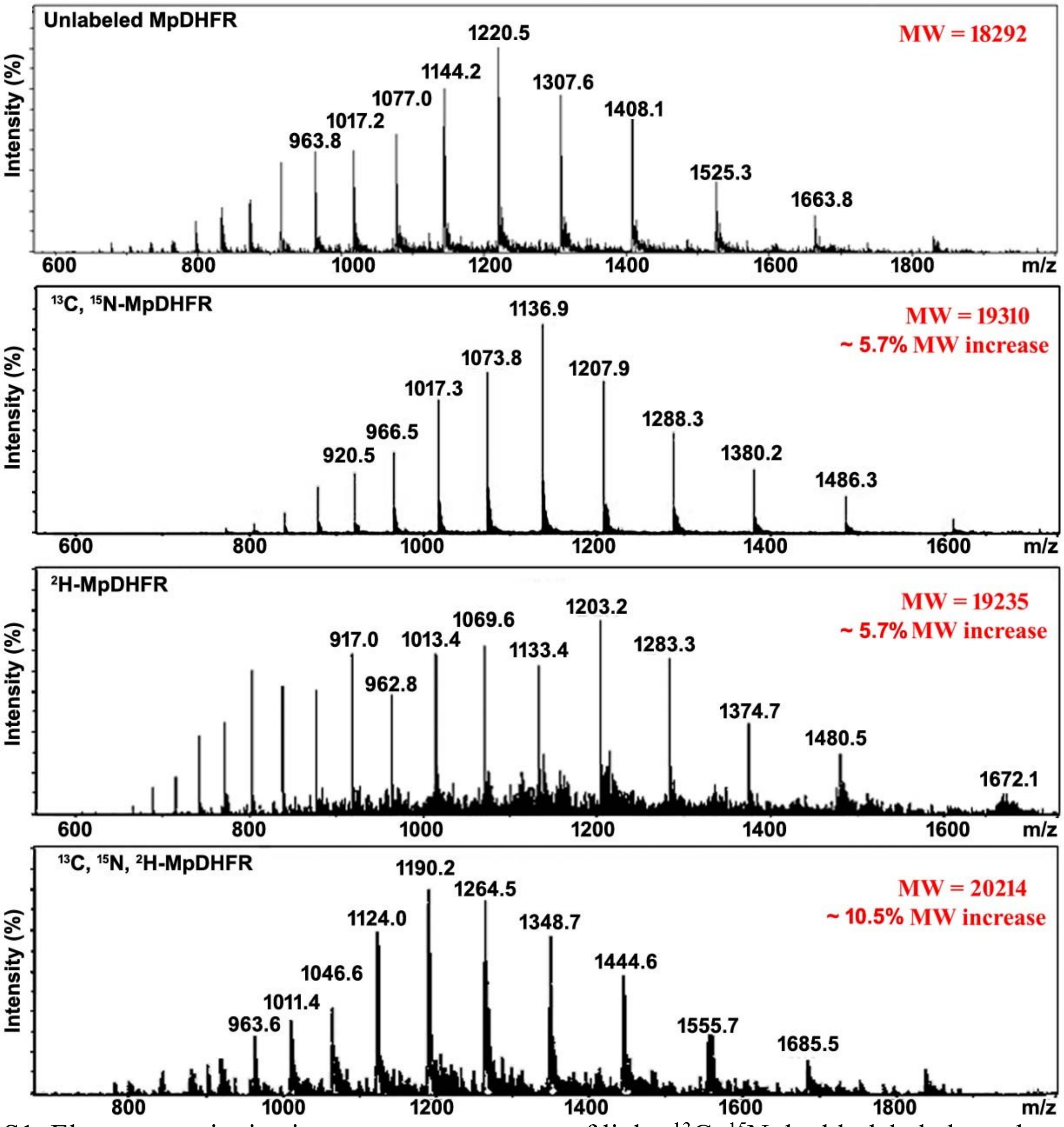

Figure S1. Electrospray ionization mass spectrometry of light, ^{13}C, ^{15}N doubly labeled, perdeuterated and ^{13}C, ^{15}N, ^{2}H-triply labeled MpDHFRs.

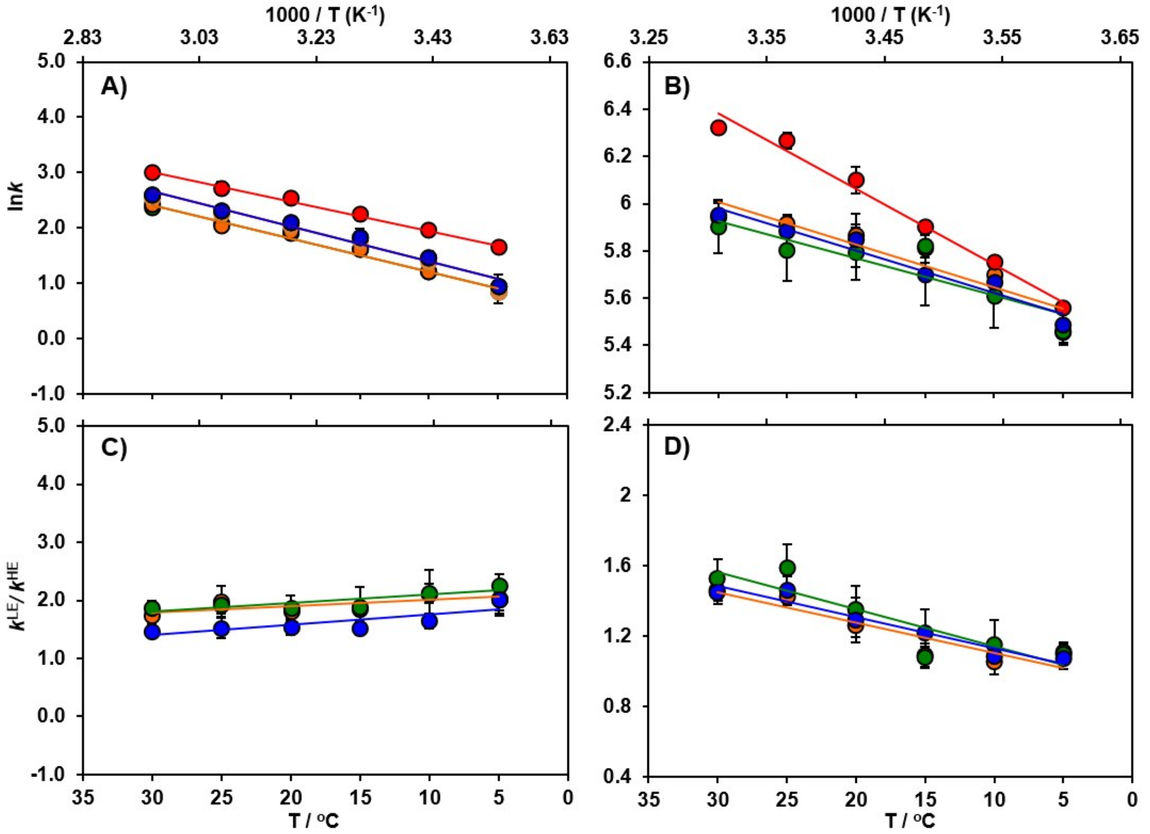

Figure S2. Arrhenius plots of the experimental A) steady-state turnover rate constants and B) pre-steady-state hydride transfer rate constants, and C-D) the temperature dependence of their corresponding enzyme KIEs (ratio of light to heavy enzyme rate constants, k^{LE}/k^{HE}, plotted on a logarithmic scale against the inverse temperature. Data for the 'light' enzyme are in red, ^{13}C, ^{15}N, ^{2}H triply labeled enzyme in blue, ^{13}C, ^{15}N doubly labeled enzyme in orange and perdeuterated enzyme in green.

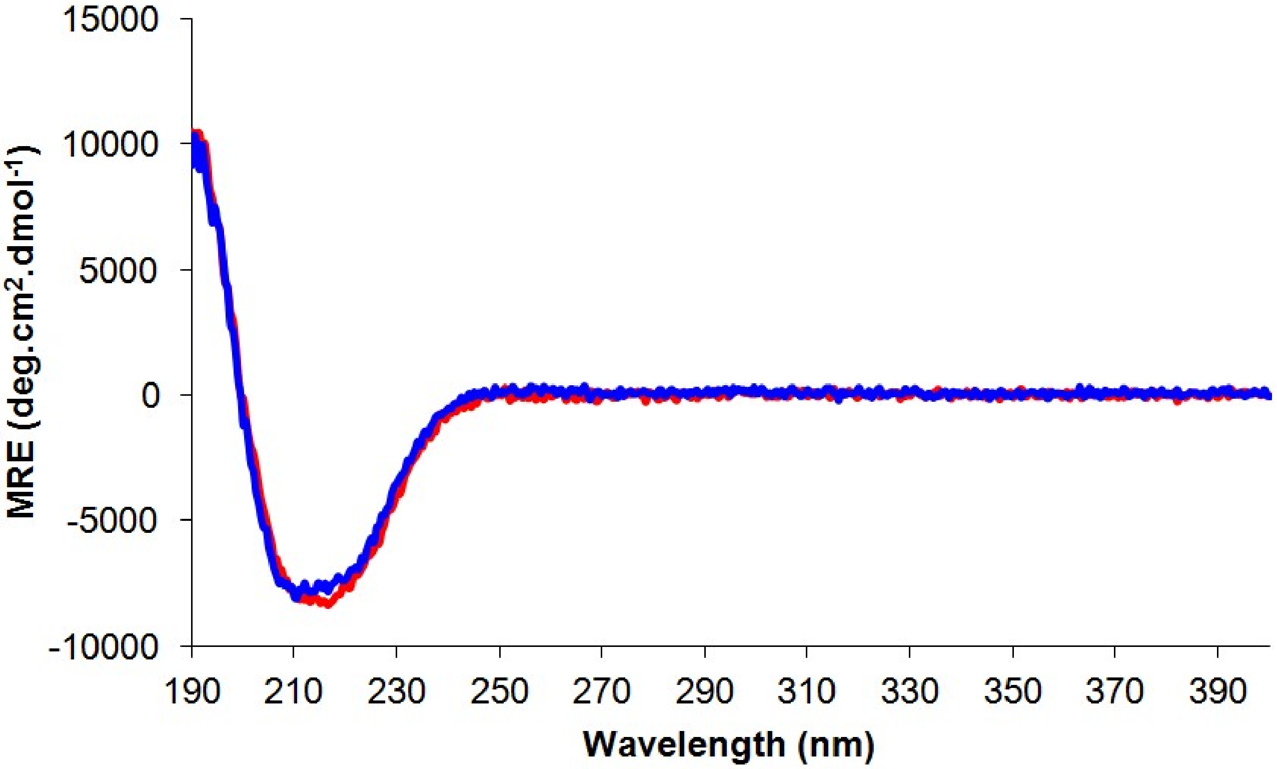

Figure S3. CD spectra for light MpDHFR (red) and ^{13}C, ^{15}N, ^{2}H triply labeled MpDHFR (blue) at 20 °C.

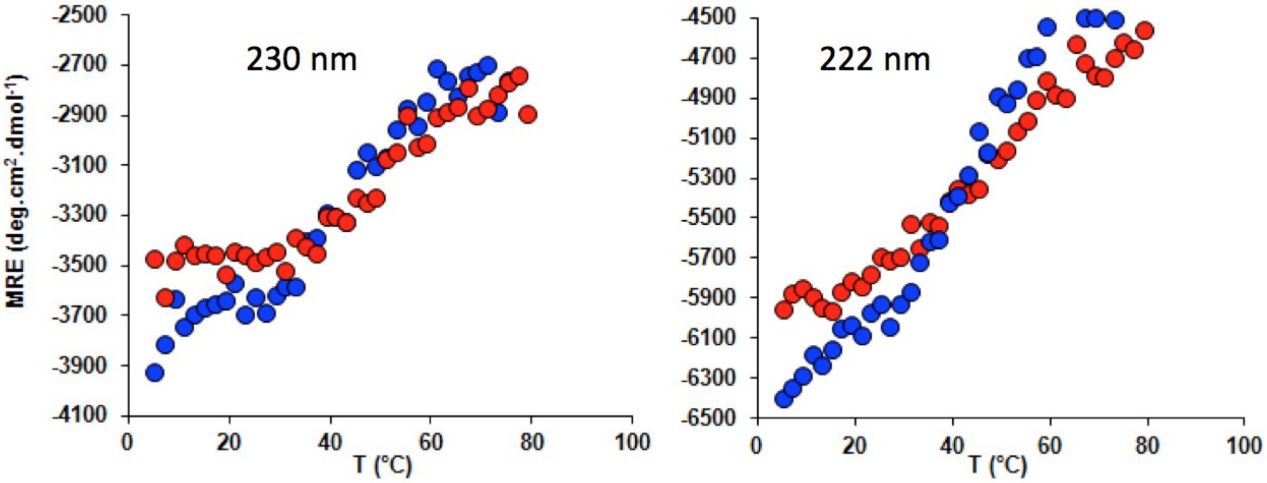

Figure S4. Change in the mean residue ellipticity at 230 nm (left) and 222 nm (right) with temperature for light (red) and ^{13}C, ^{15}N, ^{2}H triply labeled (blue) MpDHFR, measured in 10 mM potassium phosphate buffer (pH 7.0).

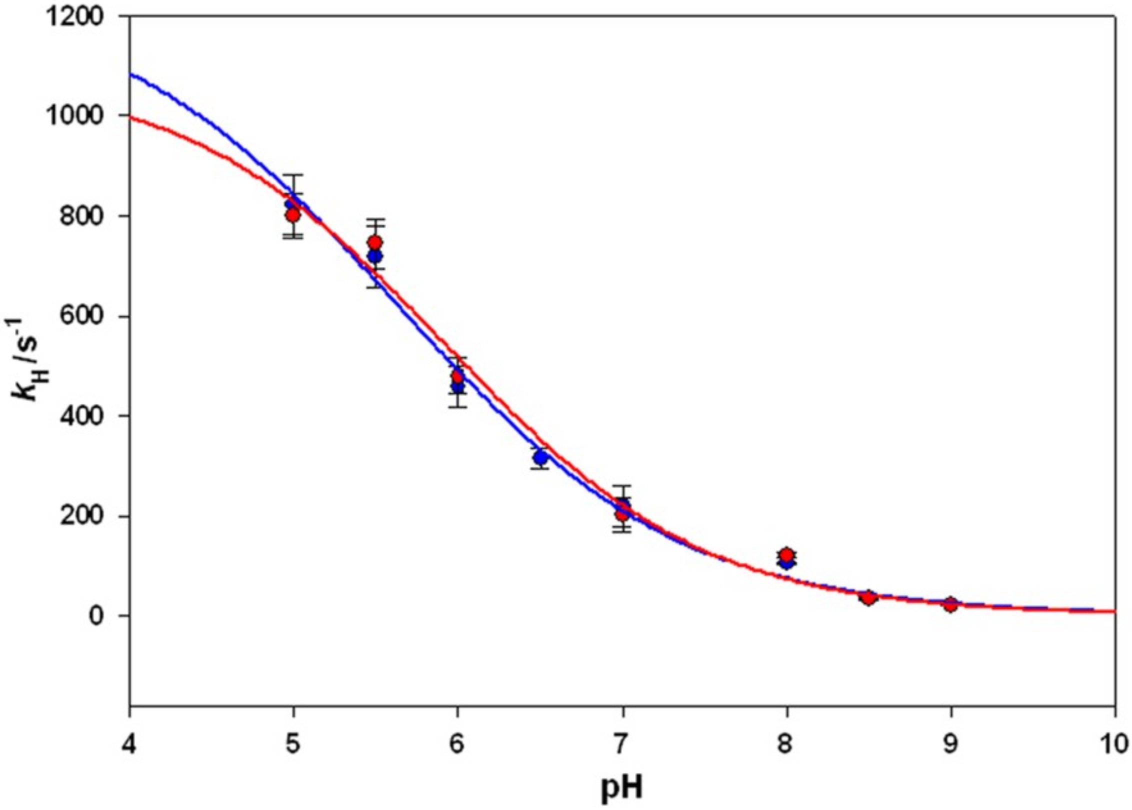

Figure S5. pH dependence of the hydride transfer rate constants for light MpDHFR (red) and ^{13}C, ^{15}N, ^{2}H triply labeled MpDHFR (blue) at 5 °C.

Table S1: Temperature dependence of the steady-state rate constant (k_{cat}) and the pre-steady-state rate constant (k_H) at pH 7.0 during catalysis by light and heavy MpDHFR.

Steady state				
T (°C)	k_{cat}^{LE} (s^{-1}) Natural abundance	k_{cat}^{HE} (s^{-1}) ^{13}C, ^{15}N, ^{2}H labeled	k_{cat}^{HE} (s^{-1}) ^{13}C, ^{15}N labeled	k_{cat}^{HE} (s^{-1}) ^{2}H labeled
5	5.20 ± 0.28	2.57 ± 0.73	2.56 ± 0.53	2.31 ± 0.42
10	7.09 ± 0.47	4.32 ± 0.51	3.34 ± 0.49	3.32 ± 1.27
15	9.39 ± 1.02	6.17 ± 0.38	5.05 ± 0.18	4.95 ± 1.57
20	12.55 ± 0.73	8.16 ± 1.04	6.89 ± 0.18	6.74 ± 1.42
25	15.24 ± 1.75	10.05 ± 1.32	7.68 ± 1.83	7.91 ± 0.87
30	19.89 ± 1.64	13.49 ± 1.05	11.40 ± 0.73	10.66 ± 1.06
Pre-steady state				
	k_H^{LE} (s^{-1}) Natural abundance	k_H^{HE} (s^{-1}) ^{13}C, ^{15}N, ^{2}H labeled	k_H^{HE} (s^{-1}) ^{13}C, ^{15}N labeled	k_H^{HE} (s^{-1}) ^{2}H labeled
5	258.86 ± 4.31	241.66 ± 13.50	233.08 ± 11.89	235.38 ± 11.49
10	314.80 ± 7.19	289.08 ± 14.63	298.67 ± 19.86	272.90 ± 36.91
15	364.32 ± 9.41	298.48 ± 38.78	334.47 ± 21.13	336.66 ± 16.23
20	445.54 ± 25.68	345.17 ± 39.45	352.67 ± 11.79	328.43 ± 38.99
25	526.50 ± 18.34	359.33 ± 24.34	369.53 ± 12.61	331.00 ± 42.43
30	556.64 ± 12.13	384.25 ± 24.06	380.83± 22.31	364.75 ± 39.95

Table S2: Temperature dependence of the heavy enzyme effect on the steady state rate constant (k_{cat}) and pre-steady state rate constant (k_H) during catalysis by MpDHFR at pH 7.0

T (°C)	Steady state $k_{cat}^{LE}/k_{cat}^{HE}$		
	^{13}C, ^{15}N, ^{2}H labeled	^{13}C, ^{15}N labeled	^{2}H labeled
5	2.02 ± 0.29	2.03 ± 0.21	2.25 ± 0.19
10	1.64 ± 0.14	2.12 ± 0.16	2.13 ± 0.39
15	1.52 ± 0.13	1.86 ± 0.11	1.90 ± 0.34
20	1.45 ± 0.14	1.82 ± 0.06	1.86 ± 0.22
25	1.52 ± 0.17	1.98 ± 0.26	1.93 ± 0.16
30	1.47 ± 0.11	1.74 ± 0.10	1.87 ± 0.13
	Pre-steady state k_H^{LE}/k_H^{HE}		
	^{13}C, ^{15}N, ^{2}H labeled	^{13}C, ^{15}N labeled	^{2}H labeled
5	1.07 ± 0.06	1.11 ± 0.05	1.10 ± 0.05
10	1.09 ± 0.06	1.05 ± 0.07	1.15 ± 0.14
15	1.22 ± 0.13	1.09 ± 0.07	1.08 ± 0.05
20	1.29 ± 0.013	1.26 ± 0.07	1.36 ± 0.13
25	1.47 ± 0.08	1.42 ± 0.05	1.59 ± 0.13
30	1.45 ± 0.07	1.46 ± 0.06	1.53 ± 0.11

Table S3: The Michaelis constants (K_M) of the light and heavy MpDHFR.

At 5 °C		
Substrate	Light	Heavy, ^{13}C, ^{15}N, ^{2}H labeled
DHF	0.70 ± 0.26	0.89 ± 0.22
NADPH	21.48 ± 8.67	21.36 ± 4.40
At 20 °C		
DHF	1.72 ± 0.32	1.78 ± 0.20
NADPH	19.11 ± 6.56	18.19 ± 4.02

Table S4: pH dependence of the pre-steady state rate constant of the light and heavy MpDHFR at 5 ºC.

pH	Light	Heavy, ^{13}C, ^{15}N, ^{2}H labeled
5	822.00 ± 59.1	800.20 ± 45.00
5.5	719.00 ± 61.6	744.50 ± 48.79
6	458.25 ± 40.00	479.80 ± 35.00
6.5	315.50 ± 20.20	Not determined
7	219.23 ± 40.00	201.55 ± 33.87
8	106.00 ± 1.41	121.00 ± 5.66
8.5	32.75 ± 0.78	35.95± 0.49
9	20.08 ± 0.6	20.65 ± 1.2

QM/MM EA-VTST calculations and molecular dynamics simulations

The simulation model. The starting structure for dynamic simulations of MpDHFR was obtained from the Protein Data Bank entry 3IA5,[4] which codes for the crystal structure of the apoenzyme. The substrate folate and cofactor NADPH were introduced based on the coordinates of the ligand-bound MpDHFR structure (PDB: 3IA4). This procedure was followed as 3IA5 has fewer unresolved atoms than 3IA4 and, particularly, the atoms of the B chain of 3IA5 are almost complete, making it the best initial model. The PROPKA3 program[5,6] was used to estimate the pK_a values of the titratable protein residues to verify their protonation states at pH 7. To neutralize the system, 11 sodium counterions were placed in optimal electrostatic positions around the enzyme. Finally, the system was solvated using a cubic box of TIP3P water molecules with side lengths of 65.0 Å; water molecules with an oxygen atom within 2.8 Å of any heavy atom were removed. The full system contained 27282 atoms, containing the protein (162 residues, 2560 atoms), the substrate and cofactor (52 and 74 atoms, respectively), 11 sodium ions and 8195 water molecules, 111 of crystallization and 8084 of solvation (a total of 24585 atoms). Heavy MpDHFR was prepared *in silico* by modifying the masses of all ^{14}N, ^{12}C and non-exchangeable ^{1}H atoms to those of ^{15}N, ^{13}C and ^{2}H, respectively. The ratio between the molecular weights of the simulated heavy and light enzymes was 1.11, similar to the experimentally measured molecular weight increase.

The whole system was divided into a QM part and a MM part to perform combined QM/MM calculations (Figure S6). The quantum subsystem contained 76 atoms, including parts of the cofactor (nicotinamide ring and the ribose) and substrate (pteridine ring and the N-methylene-substituted *p*-aminobenzoyl, pABA). Two hydrogen ‘link’ atoms[7,8] were used to saturate the valence at the QM-MM boundary (Figure S6). The quantum atoms were treated by the AM1 Hamiltonian,[9] modified using specific reaction parameters (denoted as AM1-SRP) developed previously for DHFR.[10] The protein atoms and the ions were described by OPLS-AA[11] force field while the water molecules were described by the TIP3P potential.[12] Cutoffs for the nonbonding interactions were applied using a switching function within a radius range of 13.0 to 9.0 Å. Periodic boundary conditions were employed within the minimum image convention in all the simulations.

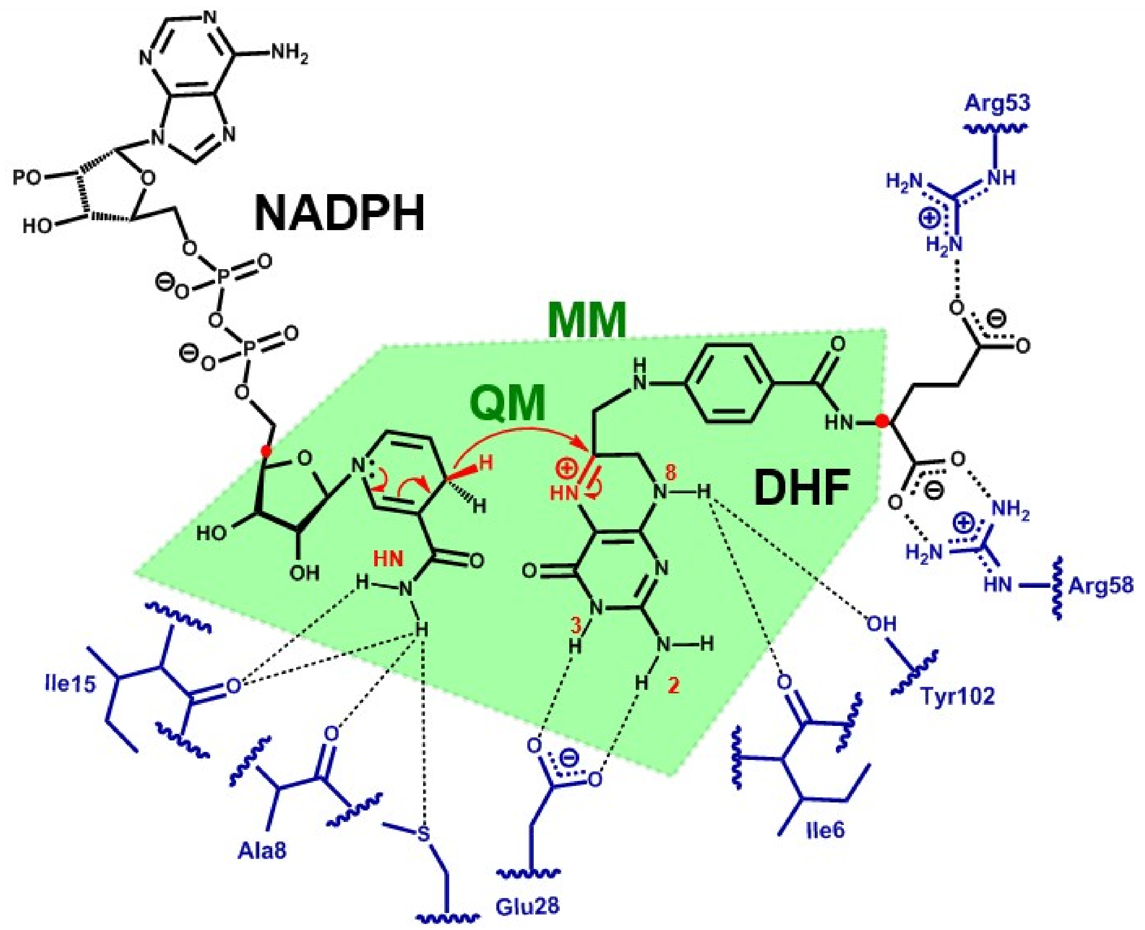

Figure S6. Schematic representation of the active site and definition of the QM/MM subsystem. QM-MM frontier link atoms are indicated as red dots.

Potential of Mean Force (PMF).[13] One-dimensional PMFs, W^{CM}, were computed using the antisymmetric combination of distances describing the hydride transfer, $\xi = d_{C4Ht} - d_{HtC6}$, as the reaction coordinate. The umbrella sampling approach was used,[14] with the system restrained to remain close to the desired value of the reaction coordinate by means of the addition of a harmonic potential with a force constant of 2500 kJ mol^{-1} A^{-2} , which allows good overlap between windows. The reaction coordinate was then explored in a range from –2.07 to 1.64 Å and the total number of windows was 53. The probability distributions obtained from MD simulations within each individual window were combined by means of the weighted histogram analysis method (WHAM).[15] 100 ps of relaxation and 100 ps of production MD, with a time step of 0.5 fs, in the canonical ensemble (NVT, with reference temperatures at 298 K) and the Langevin–Verlet integrator,[16] were used in the simulations. Three different PMFs, starting from different structures of the TS ensemble located at 298 K (Figure S7) show very small deviations between the profiles, and between the averaged structures of the three states involved in the reaction (*i.e.* all the reactant structures are similar; all the

TSs are similar to one another; and all the product states are similar to one another). It should be noted that the reaction coordinate is unaffected by the protein mass substitution, because it is defined using coordinates of the substrate and the cofactor atoms, and thus fully equilibrated PMFs will provide the same results for the heavy and light versions of the enzyme if the same force field is employed. A slight shortening of the C-H distance in the force field, mimicking the vibrational averaged reduction of the bond length observed when hydrogen is substituted by deuterium, resulted in a negligible effect on the PMF.[17]

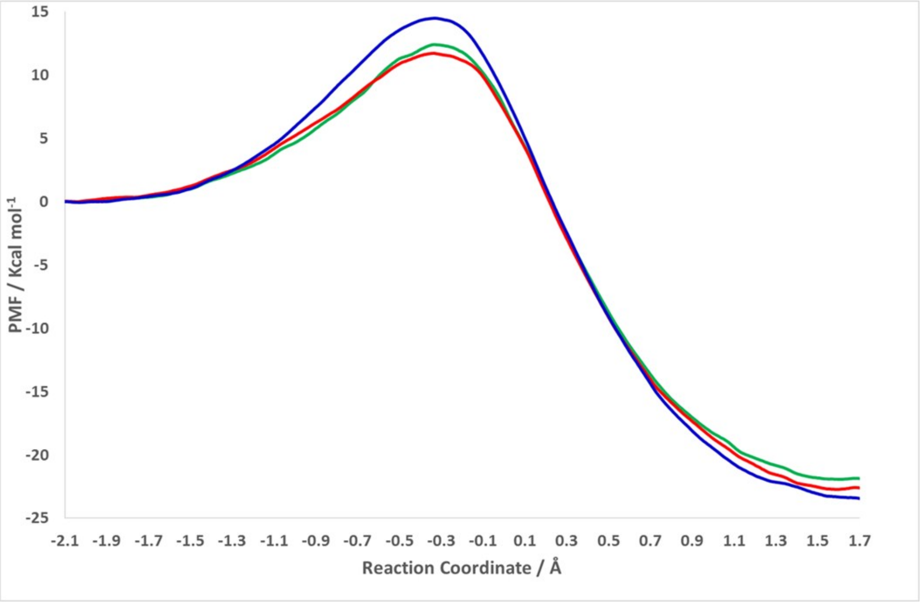

Figure S7. Classical mechanical AM1-SRP/MM Potentials of Mean Force (PMF) obtained at 298 K from different structures of the TS ensemble.

Calculation of the transmission coefficient. Grote-Hynes (GH) theory can be applied to describe the evolution of the system along the reaction coordinate at the TS. In particular, the transmission coefficient can be obtained as the ratio between the reactive frequency and the equilibrium barrier frequency:[18]

$$\gamma_{GH} = \frac{\omega_r}{\omega_{eq}} \quad \text{(S1)}$$

with the equilibrium frequency derived from a parabolic fit of the PMF around the maximum and the reactive frequency ω_r is obtained via the GH equation:[19,20]

$$\omega_r^2 - \omega_{eq}^2 + \omega_r \int_0^\infty \zeta_{TS}(t) e^{-\omega_r t} dt = 0 \quad \text{(S2)}$$

$\zeta_{TS}(t)$ is the friction kernel obtained at the TS, assuming that recrossings take place in the proximity of this dynamic bottleneck:[20,21]

$$\zeta(t) = \frac{\langle F_{RC}(0)F_{RC}(t)\rangle}{\mu_{RC} k_B T} \tag{S3}$$

where $F_{RC}(t)$ is the force projected on the reaction coordinate (the antisymmetric combination of the distances of the hydride to the donor and acceptor) and μ_{RC} the associated reduced mass. The friction kernel was averaged from the force autocorrelation, obtained from MD simulations carried out with the reaction coordinate fixed at the maximum of the PMF. For the evaluation of the TS friction kernel at different temperatures, the system was first equilibrated at the reactants state by means of 5 ns of classical MD at 278, 283, 288, 293, 298, 303 and 308 K using NAMD[22] software. Then by means of QM/MM MD simulations, we determined the one dimensional PMF in the vicinity of the transition state region at each temperature using the fDYNAMO library.[23] 50 ps (10 ps of relaxation and 40 ps of production) of constrained QM/MM MD simulations were run at windows of the PMFs. Finally, a QM/MM MD simulation of 20 ps was performed from the top of the PMF (fixing the value of the reaction coordinate) with a small time step of 0.1 fs to ensure the convergence of the algorithm and forces acting on the reaction coordinate were saved at each simulation step. We previously tested that the GH approach gives transmission coefficients in very good agreement with those obtained from activated trajectories initiated at the TS ensemble.[24,25]

The recrossing transmission coefficients $\gamma(T,\xi)$ were calculated using eq. S1 for the light and heavy versions of MpDHFR. Because the heavy enzyme is slower it presents a higher friction and thus a smaller value of the transmission coefficient. The values obtained at the different temperatures are presented in Table S5. The transmission coefficients of the two versions were found to be statistically different.

Table S5: Transmission coefficients of light and heavy MpDHFR and corresponding KIEs evaluated using GH theory.

Temperature	Light	Heavy, ^{13}C, ^{15}N, ^{2}H labeled	KIE
278	0.67 ± 0.02	0.65 ± 0.01	1.03 ± 0.03
283	0.67 ± 0.03	0.61 ± 0.02	1.10 ± 0.06
288	0.70 ± 0.01	0.62 ± 0.01	1.13 ± 0.02
293	0.66 ± 0.03	0.56 ± 0.03	1.18 ± 0.07
298	0.59 ± 0.03	0.48 ± 0.02	1.23 ± 0.07
303	0.57 ± 0.03	0.46 ± 0.02	1.24 ± 0.07
308	0.57 ± 0.01	0.44 ± 0.01	1.30 ± 0.03

Ensemble Averaged Variational Transition State Theory. Deviations from classical Transition State Theory (TST) as a result of quantum tunneling effects can be estimated by means of Ensemble-Averaged Variational Transition State Theory (EA-VTST).[26-28] In this approach, the theoretical estimation of the rate constant can be written as:

$$k(T) = \Gamma(T,\xi)\frac{k_B T}{h} e^{-\left(\frac{\Delta G_{act}^{QC}(T,\xi)}{RT}\right)} \quad \text{(S4)}$$

ΔG_{act}^{QC} is the quasiclassical activation free energy at the transition state, obtained from the classical mechanical (CM) PMF and included a correction for quantizing the vibrations orthogonal to the reaction coordinate and the vibrational free energy of the reactant mode that correlates with motion along the reaction coordinate, and is calculated as:

$$\Delta G_{act}^{QC} = [W^{CM}(T,\xi^*) + \Delta W_{vib}(T,\xi^*)] - [W^{CM}(T,\xi_R) + \Delta W_{vib,R}(T) + G_{R,T,F}^{CM}] \quad \text{(S5)}$$

where $\Delta W_{vib}(T,\xi^*)$ corrects $W^{CM}(T,\xi^*)$ to account for quantized vibrations orthogonal to the reaction coordinate along which the PMF is defined, *z,* at the maximum of the PMF, ξ^*; $\Delta W_{vib,R}(T)$ corrects $W^{CM}(T,\xi_R)$ for quantized vibrations at the reactant side minimum of the PMF, ξ_R, and $G^{CM}_{R,T,F}$ is a

correction for the vibrational free energy of the reactant mode that correlates with motion along the reaction coordinate.[26]

To correct the classical mechanical PMF, W^{CM}, normal mode analyses were performed for the quantum region atoms. ΔG_{act}^{QC} is obtained by equation (S4), with the terms defined above. To perform these calculations, in addition to the PMFs obtained as described above, we localized 15 TS structures starting from different configurations of the corresponding simulation windows in the heavy and light enzymes. After tracing the minimum energy path, we optimized 15 reactant structures and obtained the Hessian matrix for all the stationary structures.[29-31] The final quantum mechanical vibrations correction to the quasi-classical activation free energy was obtained as an average over these structures.

The transmission coefficient, Γ, is obtained as the product of recrossing (γ) and tunneling (κ) contributions:

$$\Gamma\,(T,\xi) = \gamma\,(T,\xi)\cdot\kappa(T) \qquad \text{(S6)}$$

The recrossing transmission coefficients for the heavy and light versions of the enzyme, γ, were calculated as described above (Eq (S1)). The tunneling transmission coefficients, κ, were calculated with the small-curvature tunneling (SCT) approximation, which includes reaction-path curvature appropriate for enzymatic hydride transfers and, in particular, for DHFR.[29-32] The final tunneling contribution (see main text) is obtained as the average over the reaction paths of 15 TS structures.

Finally, Eq (S4) can be transformed into Eq (S6) by incorporating the transmission coefficient into the exponential term, giving a phenomenological free energy of activation, ΔG_{eff}:

$$k(T) = \frac{k_B T}{h} e^{-\left(\frac{\Delta G_{eff}(T,\xi)}{RT}\right)} \qquad \text{(S7)}$$

Table S6: Key averaged structural parameters of the reactant state, RS, and transition state, TS, from the PMFs of MpDHFR obtained at 298 K. Distances (d) are in Å and angles in degrees.

	$RS_{classical}$	RS_{QMMM}	TS_{QMMM}
dC_{don}-H_{trans}	1.09 ± 0.03	1.11 ± 0.03	1.37 ± 0.04
dC_{don}-C_{acc}	4.0 ± 0.4	4.03 ± 0.01	2.65 ± 0.06
dC_{acc}-H_{trans}	3.1 ± 0.4	3.02 ± 0.04	1.31 ± 0.03
Angle C_{don}-H_{trans}-C_{acc}	142 ± 16	154 ± 12	165 ± 6
$dH_{cotrans}$-O_{TYR102}	2.9 ± 0.3	3.1 ± 0.4	2.6 ± 0.2
$dN8_{folate}$-O_{TYR102}	4.0 ± 0.4	3.7 ± 0.2	3.9 ± 0.4
$dHN1_{cof}$-O_{ALA8}	2.6 ± 0.4	3.3 ± 1.3	2.6 ± 0.7
$dHN1_{cof}$-O_{ILE15}	3.7 ± 0.4	4.4 ± 1.3	4.8 ± 0.5
$dHN2_{cof}$-O_{ALA8}	3.8 ± 0.3	4.3 ± 0.6	3.2 ± 0.8
$dHN2_{cof}$-O_{ILE15}	2.4 ± 0.4	4.0 ± 0.7	4.7 ± 0.5
$dHN8_{folate}$ -O_{ILE6}	2.9 ± 0.7	2.5 ± 0.3	2.5 ± 0.3
$dHN2_{cof}$–SD_{MET21}	4.5 ± 0.6	5.8 ± 0.7	6.8 ± 0.9

Table S7: Key averaged structural parameters of the reactant state, RS, from the 5 ns of classical MD of MpDHFR obtained at 278, 298 and 308 K. Distances (d) are in Å and angle in degrees.

	T = 278 K	***T = 298 K***	***T = 308 K***
dC_{don}-H_{trans}	1.09 ± 0.03	1.09 ± 0.03	1.09 ± 0.03
dC_{don}-C_{acc}	4.5 ± 0.4	4.0 ± 0.4	4.2 ± 0.4
dC_{acc}-H_{trans}	3.7 ± 0.4	3.1 ± 0.4	3.5 ± 0.5
Angle C_{don}-H_{trans}-C_{acc}	142 ± 17	142 ± 16	124 ± 27
$dHN_{cotrans}$-O_{TYR102}	3.4 ± 0.6	2.9 ± 0.3	2.9 ± 0.6
$dN8_{folate}$-O_{TYR102}	4.1 ± 0.8	4.0 ± 0.4	4.3 ± 0.4
$dHN1_{cof}$-O_{ALA8}	2.6 ± 0.4	2.6 ± 0.4	2.2 ± 0.4
$dHN1_{cof}$-O_{ILE15}	3.8 ± 0.5	3.7 ± 0.4	5.0 ± 0.8
$dHN2_{cof}$-O_{ALA8}	3.7 ± 0.4	3.8 ± 0.3	3.6 ± 0.3
$dHN2_{cof}$-O_{ILE15}	2.6 ± 0.6	2.4 ± 0.4	4.1 ± 0.8
$dHN8_{folate}$-O_{ILE6}	3.2 ± 0.7	2.9 ± 0.7	3.1 ± 0.6
$dHN2_{cof}$ –SD_{MET21}	4.3 ± 0.7	4.5 ± 0.6	5.2 ± 1.1

```
MpDHFR          MIVSMIAALANNRVIGLDNKMPWHLPAELQLFKRATLGKP-IVMGRNTFESIGRPLPGRL 59
EcDHFR          -MISLIAALAVDRVIGMENAMPWNLPADLAWFKRNTLDKP-VIMGRHTWESIGRPLPGRK 58
BSDHFR          -MISHIVAMDENRVIGKDNRLPWHLPADLAYFKRVTMGHA-IVMGRKTFEAIGRPLPGRD 58
TmDHFR          AKVIFVLAMDVSGKIAS-SVESWSSFEDRKNFRKITTEIGNVVMGRITFEEIGRPLPERL 59
                  :  : *:  .  *.  .  .*    :   *:: *     ::*** *:* ****** *

MpDHFR          NIVLSRQTDYQPEGVTVVAT---LEDAVVAAG-DVEELMIIGGATIYNQCL--AAADRLY 113
EcDHFR          NIILSSQPG-TDDRVTWVKS---VDEAIAACG-DVPEIMVIGGGRVYEQFL--PKAQKLY 111
BSDHFR          NVVVTGNRSFRPEGCLVLHS---LEEVKQWIASRADEVFIIGGAELFRATM--PIVDRLY 113
TmDHFR          NVVLTRRPKTSNNPSLVFFNGSPADVVKFLEGKGYERVAVIGGKTVFTEFLREKLVDELF 119
                *:::: .     :    . .    : .    .    .: :***  ::   :    .:.*:

MpDHFR          LTHIELTTEGDTWFPDYEQYNWQEIEHESYAADDKNPHNYRFSLLERVK 162
EcDHFR          LTHIDAEVEGDTHFPDYEPDDWESVFSEFHDADAQNSHSYCFEILERR- 159
BSDHFR          VTKIFASFPGDTFYPPISDDEWEIVSYTPGGKDEKNPYEHAFIIYERK- 161
TmDHFR          VTVEPYVFGKGIPFFDEFEGYFPLKLLEMRRLNERGTLFLKYSVEKSHR 168
                :*        .  :       :          : :..    : : :
```

Table S8: Sequence identity and similarity scores for MpDHFR, EcDHFR, BsDHFR and TmDHFR		
Enzymes	Identity (%)	Similarity (%)
Mp and Ec	53.70	17.28
Mp and Bs	38.51	21.74
Mp and Tm	23.67	20.12
Ec and Bs	42.94	23.93
Ec and Tm	21.89	20.12
Bs and Tm	24.85	16.57

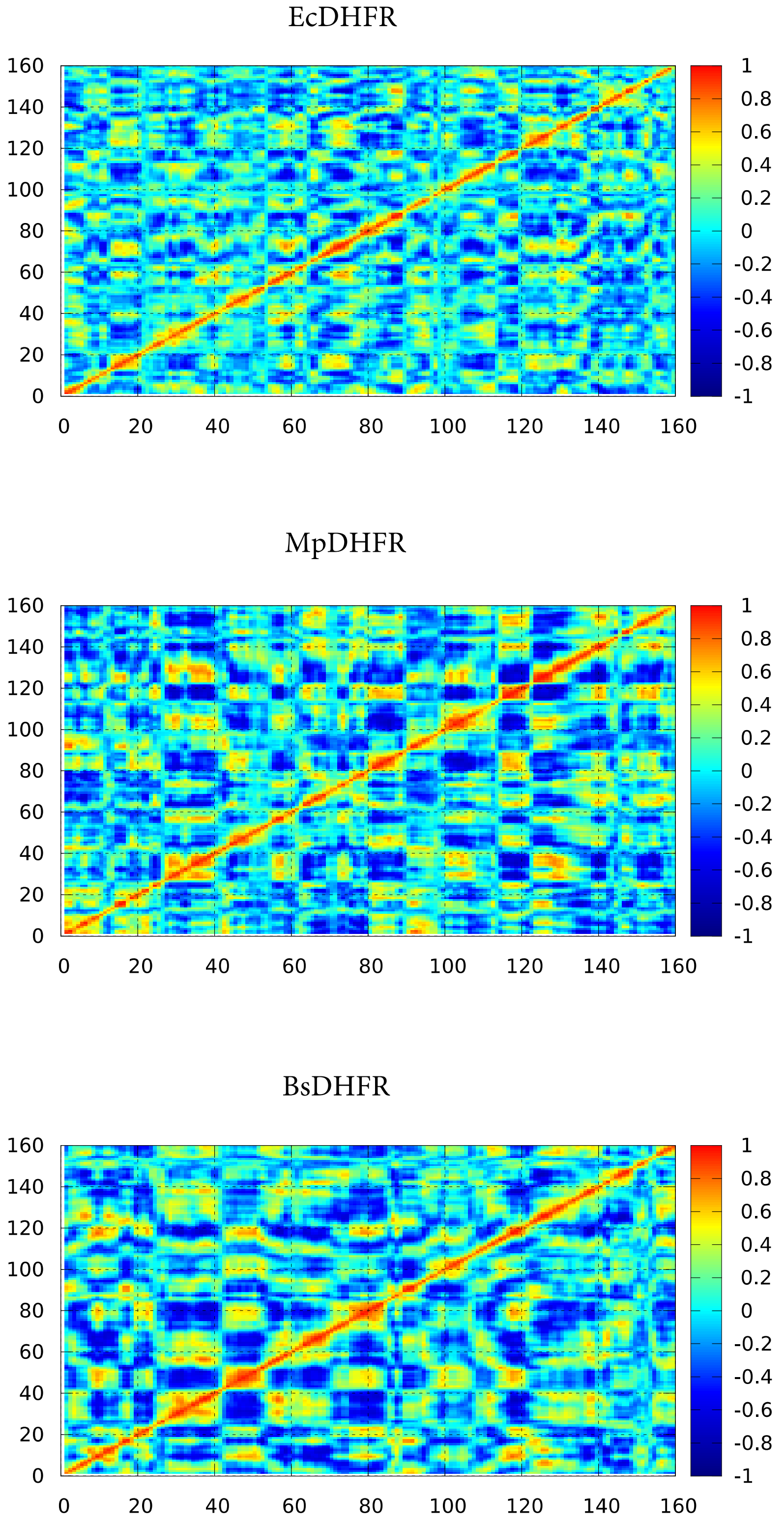

Figure S8. Dynamic cross-correlation map (DCCM)[33], measuring the correlation between the displacements of Cα atoms, calculated from QM/MM simulations of the reactants state of EcDHFR, MpDHFR and BsDHFR. The correlation scores are encoded with a colour gradient from −1 (blue, completely anti-correlated) to +1 (red, completely correlated).

References

(1) Birdsall, B.; Burgen, A. S. V.; Roberts, G. C. K. *Biochemistry* **1980**, *19*, 3723.

(2) Luk, L. Y. P.; Ruiz-Pernia, J. J.; Dawson, W. M.; Loveridge, E. J.; Tunon, I.; Moliner, V.; Allemann, R. K. *J Am Chem Soc* **2014**, *136*, 17317.

(3) Loveridge, E. J.; Matthews, S. M.; Williams, C.; Whittaker, S. B. M.; Gunther, U. L.; Evans, R. M.; Dawson, W. M.; Crump, M. P.; Allemann, R. K. *Biomol Nmr Assign* **2013**, *7*, 61.

(4) Hay, S.; Evans, R. M.; Levy, C.; Loveridge, E. J.; Wang, X.; Leys, D.; Allemann, R. K.; Scrutton, N. S. *Chembiochem : a European journal of chemical biology* **2009**, *10*, 2348.

(5) Sondergaard, C. R.; Olsson, M. H. M.; Rostkowski, M.; Jensen, J. H. *J Chem Theory Comput* **2011**, *7*, 2284.

(6) Olsson, M. H. M.; Sondergaard, C. R.; Rostkowski, M.; Jensen, J. H. *J Chem Theory Comput* **2011**, *7*, 525.

(7) Singh, U. C.; Kollman, P. A. *J Comput Chem* **1986**, *7*, 718.

(8) Field, M. J.; Bash, P. A.; Karplus, M. *J Comput Chem* **1990**, *11*, 700.

(9) Dewar, M. J. S.; Zoebisch, E. G.; Healy, E. F.; Stewart, J. J. P. *J Am Chem Soc* **1985**, *107*, 3902.

(10) Doron, D.; Major, D. T.; Kohen, A.; Thiel, W.; Wu, X. *J Chem Theory Comput* **2011**, *7*, 3420.

(11) Jorgensen, W. L.; Tiradorives, J. *J Am Chem Soc* **1988**, *110*, 1657.

(12) Jorgensen, W. L.; Chandrasekhar, J.; Madura, J. D.; Impey, R. W.; Klein, M. L. *J Chem Phys* **1983**, *79*, 926.

(13) Roux, B. *Comput Phys Commun* **1995**, *91*, 275.

(14) Torrie, G. M.; Valleau, J. P. *J Comput Phys* **1977**, *23*, 187.

(15) Kumar, S.; Bouzida, D.; Swendsen, R. H.; Kollman, P. A.; Rosenberg, J. M. *J Comput Chem* **1992**, *13*, 1011.

(16) Paterlini, M. G.; Ferguson, D. M. *Chem Phys* **1998**, *236*, 243.

(17) Luk, L. Y. P.; Ruiz-Pernía, J. J.; Dawson, W. M.; Loveridge, E. J.; Tuñón, I.; Moliner, V.; Allemann, R. K. *J. Am. Chem. Soc.* **2014**, *136*, 17317.

(18) Gertner, B. J.; Wilson, K. R.; Hynes, J. T. *J Chem Phys* **1989**, *90*, 3537.

(19) Grote, R. F.; Hynes, J. T. *J Chem Phys* **1980**, *73*, 2715.

(20) Hynes, J. T. *The Theory of Chemical Reaction Dynamics* Boca Raton, FL, 1985; Vol. IV.

(21) Kim, H. J.; Hynes, J. T. *J Am Chem Soc* **1992**, *114*, 10508.

(22) Phillips, J. C.; Braun, R.; Wang, W.; Gumbart, J.; Tajkhorshid, E.; Villa, E.; Chipot, C.; Skeel, R. D.; Kale, L.; Schulten, K. *J Comput Chem* **2005**, *26*, 1781.

(23) Field, M. J.; Albe, M.; Bret, C.; Proust-De Martin, F.; Thomas, A. *J Comput Chem* **2000**, *21*, 1088.

(24) Roca, M.; Oliva, M.; Castillo, R.; Moliner, V.; Tunon, I. *Chem-Eur J* **2010**, *16*, 11399.

(25) Luk, L. Y. P.; Ruiz-Pernia, J. J.; Dawson, W. M.; Roca, M.; Loveridge, E. J.; Glowacki, D. R.; Harvey, J. N.; Mulholland, A. J.; Tunon, I.; Moliner, V.; Allemann, R. K. *P Natl Acad Sci USA* **2013**, *110*, 16344.

(26) Alhambra, C.; Corchado, J.; Sanchez, M. L.; Garcia-Viloca, M.; Gao, J.; Truhlar, D. G. *J Phys Chem B* **2001**, *105*, 11326.

(27) Truhlar, D. G.; Gao, J. L.; Alhambra, C.; Garcia-Viloca, M.; Corchado, J.; Sanchez, M. L.; Villa, J. *Accounts Chem Res* **2002**, *35*, 341.

(28) Truhlar, D. G.; Gao, J. L.; Garcia-Viloca, M.; Alhambra, C.; Corchado, J.; Sanchez, M. L.; Poulsen, T. D. *Int J Quantum Chem* **2004**, *100*, 1136.

(29) Pu, J. Z.; Gao, J. L.; Truhlar, D. G. *Chemical Reviews* **2006**, *106*, 3140.

(30) Pang, J. Y.; Pu, J. Z.; Gao, J. L.; Truhlar, D. G.; Allemann, R. K. *J Am Chem Soc* **2006**, *128*, 8015.

(31) Garcia-Viloca, M.; Truhlar, D. G.; Gao, J. L. *Biochemistry-Us* **2003**, *42*, 13558.
(32) Ruiz-Pernia, J. J.; Luk, L. Y. P.; Garcia-Meseguer, R.; Marti, S.; Loveridge, E. J.; Tunon, I.; Moliner, V.; Allemann, R. K. *J Am Chem Soc* **2013**, *135*, 18689.
(33) Ichiye, T.; Karplus, M. *Proteins: Structure, Function, and Bioinformatics* **1991**, *11*, 205.
